# Enablers and Inhibitors to Implementing Tobacco Cessation Interventions within Homeless-Serving Agencies: A Qualitative Analysis of Program Partners’ Experiences

**DOI:** 10.3390/cancers16112162

**Published:** 2024-06-06

**Authors:** Isabel Martinez Leal, Ammar D. Siddiqi, Anastasia Rogova, Maggie Britton, Tzuan A. Chen, Teresa Williams, Kathleen Casey, Hector Sanchez, Lorraine R. Reitzel

**Affiliations:** 1 Department of Behavioral Science, The University of Texas MD Anderson Cancer Center, 1155 Pressler Street, Houston, TX 77030, USA; asiddiqi1@mdanderson.org (A.D.S.); mbritton@mdanderson.org (M.B.); hsanchez1@mdanderson.org (H.S.); lreitzel@mdanderson.org (L.R.R.); 2 Department of Biosciences, Rice University, 6100 Main Street, Houston, TX 77005, USA; 3 Department of Health Services Research, The University of Texas MD Anderson Cancer Center, 1400 Pressler Street, Houston, TX 77030, USA; arogova@mdanderson.org; 4 Department of Psychological, Health & Learning Sciences, The University of Houston, 3657 Cullen Blvd., Houston, TX 77204, USA; tchen3@central.uh.ed; 5 Health Research Institute, The University of Houston, 4349 Martin Luther King Blvd., Houston, TX 77204, USA; 6 Integral Care, 1430 Collier Street, Austin, TX 78704, USA; teresa.williams@integralcare.org (T.W.); kathleen.casey@integralcare.org (K.C.)

**Keywords:** tobacco-related cancers, tobacco dependence, prevention research, people experiencing homelessness, tobacco-free workplace interventions, qualitative research

## Abstract

**Simple Summary:**

People experiencing homelessness are at increased risk of dying from tobacco-related cancers due to their elevated tobacco use rates but are not offered evidence-based tobacco dependence interventions by homeless-serving agencies within the United States. Through pre- and post-implementation provider interviews, this qualitative study explored the factors enabling and inhibiting organizational readiness to implement a comprehensive tobacco-cessation intervention within three homeless-serving agencies. Although the organizational readiness was initially high, at the post-implementation, changing contextual factors, primarily resource privations, undermined the provider change efficacy and limited the program implementation. These findings support the value and acceptability of implementing tobacco-cessation interventions within homeless-serving agencies, and they identify the factors needed to build organizational capacity for successful implementation.

**Abstract:**

Despite the high tobacco use rates (~80%) and tobacco-related cancers being the second leading cause of death among people experiencing homelessness within the United States, these individuals rarely receive tobacco use treatment from homeless-serving agencies (HSAs). This qualitative study explored the enablers and inhibitors of implementing an evidence-based tobacco-free workplace (TFW) program offering TFW policy adoption, specialized provider training to treat tobacco use, and nicotine replacement therapy (NRT) within HSAs. Pre- and post-implementation interviews with providers and managers (*n* = 13) pursued adapting interventions to specific HSAs and assessed the program success, respectively. The organizational readiness for change theory framed the data content analysis, yielding three categories: change commitment, change efficacy and contextual factors. Pre- to post-implementation, increasing challenges impacted the organizational capacity and providers’ attitudes, wherein previously enabling factors were reframed as inhibiting, resulting in limited implementation despite resource provision. These findings indicate that low-resourced HSAs require additional support and guidance to overcome infrastructure challenges and build the capacity needed to implement a TFW program. This study’s findings can guide future TFW program interventions, enable identification of agencies that are well-positioned to adopt such programs, and facilitate capacity-building efforts to ensure their successful participation.

## 1. Introduction

Tobacco use remains the leading cause of preventable death, disease, and disability in the United States (US) [1] and has been linked to at least 13 different cancers, accounting for 30% of all cancer-related deaths [2]. Although recent tobacco control efforts have been successful in reducing the overall prevalence of smoking, immense disparities persist in certain subpopulations [3]. Among adults experiencing homelessness, the estimated prevalence of smoking is as high as 80%, which is seven-fold greater than that of the general population in the US [4]. According to the US Department of Housing and Urban Development, in 2023, the national rate of those experiencing homelessness increased 12% over 2022, rising to 19.6 people out of every 10,000 in the general population; the greatest number measured within the 18-year history of this survey [5]. The environmental and behavioral risk factors for developing cancer are high for these individuals [6]. Prior research among homeless adults has shown cancer to be the second overall cause of death and the leading cause among those 45 years or older [6]. Furthermore, the concurrent use of multiple tobacco products is estimated at between 51.1% and 68% in some studies [7,8,9], which is dramatically greater than the national prevalence of 17% [10]. Despite the disproportionate use of tobacco products among individuals experiencing homelessness, they are significantly less likely to be advised to quit or have a successful quit attempt, thereby preserving existing disparities in the cancer incidence and mortality [11].

Addressing the elevated use of tobacco and its adverse health effects among individuals experiencing homelessness remains challenging for several reasons. One significant challenge stems from the fact that nearly 60% of individuals in this population lack health insurance [12]. As a result, they face structural barriers to accessing crucial smoking cessation therapies and professional healthcare guidance to support their quit attempts. Additionally, the perception of tobacco use as a means of reducing chronic stress from being unsheltered [13] and helping to cope with psychiatric disorders [14] is pervasive in this population and contributes to the low levels of self-efficacy when attempting to quit [15]. Moreover, individuals experiencing homelessness often experience significant barriers to accessing cancer screenings, which are critical for early detection, successful cancer-related health outcomes, and survival. However, the aforementioned barriers can be alleviated by disseminating information about free health resources (e.g., quitlines) [16], given the prevalence of cell phone usage and the potential utility of mobile health technologies among adults experiencing homelessness [17]. Other available resources include non-profits offering free cancer screenings and disseminating scientific findings establishing that tobacco use worsens stress [18] and can exacerbate psychiatric disorders [19] to correct misperceptions in this group.

The prevalence of tobacco usage among individuals experiencing homelessness is further elevated by the lack of prioritization of treating tobacco dependence in agencies where they receive assistance (e.g., shelters) [20]. Many agencies serving those who are unhoused, although dedicated to helping them, have not implemented a tobacco-free workplace (TFW) policy that disallows tobacco use on-site or limits it to designated areas [20,21]. This is despite the fact that prior work supports sizeable client interest (32–64%) in having smoke-free policies in these settings [22,23]. While agency administrators’ reluctance to implement even partial TFW policies can stem from valid concerns that doing so will keep those experiencing homelessness from seeking services, research does not support a lowering of occupancy rates following policy adoption [24]. Moreover, past-year education receipt on the hazards of smoking, including tobacco-related cancer morbidity and mortality, has been reported to be as low as 20% among providers in these settings [25]. Consequently, these agencies unintentionally foster an environment where tobacco use is not only tolerated but accepted. These norms have far-reaching negative consequences, contributing to salient behaviors observed within this setting, including providers smoking with their clients and encouraging clients to use tobacco as a coping mechanism [26,27], thus actively discouraging cessation [20]. Furthermore, they may affect client care receipt in this setting by contributing to reduced delivery of nicotine replacement therapy (NRT) and behavioral interventions that facilitate tobacco cessation [28] and are critical for cancer prevention. Thus, it is imperative for homeless-serving agencies to cultivate an environment that encourages their client population to quit tobacco.

Extensive research has shown that the implementation of evidence-based TFW programs can yield significant improvements in tobacco care delivery in this setting [23,24,29,30,31,32,33,34] to address cancer-related health disparities. Providers who participate in such programs report notable gains in tobacco-related knowledge and receipt of training on the hazards of smoking (e.g., cancer risk) [25]. Additionally, these programs enhance the use of tobacco interventions, as demonstrated by the mean increases in providers’ use of the 5As brief intervention for tobacco use (ask, advise, assess, assist, and arrange), delivery of pharmacotherapy, and non-nicotine medications. As a result of increased access to evidence-based interventions, individuals experiencing homelessness are more likely to decrease their smoking frequency [35,36] and report a higher percentage of smoking abstinence over time [37], which can significantly contribute to reducing their risk of developing cancers. Notably, implementation of TFW programs to achieve gains in tobacco care provision is facilitated by organizational readiness or capacity to deliver evidence-based tobacco cessation interventions in various settings, including substance use and mental health treatment centers [32,38,39,40]. Prior findings underscore the value of TFW programs and the role of agency characteristics in facilitating program adoption.

However, the implementation of a TFW program is not without its challenges. Qualitative studies have consistently reported that the acceptance of tobacco use on-site, both by clients and by staff, poses a significant barrier to the adoption of partial or full tobacco-free policy programs [40] that promote cancer prevention. Behaviors contributing to these barriers include staff not having tobacco intervention training [41], the ubiquity of tobacco on-site [42], and staff perceiving tobacco dependence as unimportant [43]. Additionally, wider implementation is often precluded by staff turnover, limited group coordination, and provider reports of inadequate time to address clients’ tobacco dependence [44]. It is worth noting that many of these barriers are influenced by how willing an organization is to implement change to address client and staff tobacco dependence. As the barriers to addressing tobacco dependence within agencies serving the unhoused, like those treating individuals with substance use disorders, are predominantly systemic in nature, researchers recommend implementing interventions focused on organizational change [34,45,46,47]. Therefore, further investigations that assess organizational readiness contributions to program efficacy are warranted.

There is limited research available on the factors and processes influencing successful implementation of tobacco cessation interventions within homeless-serving agencies to combat the disproportionate burden of cancer incidence, mortality, and survivorship in the population they serve [6,25,36,48,49]. Despite the available practice guidelines [50,51] recommending evidence-based tobacco cessation interventions for adults experiencing homelessness, who additionally disproportionately suffer from psychiatric disorders, uptake of these practices within homeless-serving agencies has been limited [50]. This represents a valuable missed opportunity, as these individuals have limited access to evidence-based tobacco cessation interventions [11,52], such as behavioral health counseling and pharmacotherapy, due to their lack of stable housing, employment, and consequently, medical insurance [53]. Additionally, individuals experiencing homelessness encounter significant cessation barriers, including suffering from high rates of post-traumatic stress disorder, which research indicates is positively associated with smoking [54]. Unfortunately, research documents that staff within homeless-serving agencies do not support clients in making quit attempts [21] or provide consistent access to cessation interventions [49,55]. The fact that evidence-based tobacco cessation services are not consistently offered within homeless-serving agencies signals the need to identify the implementation barriers to their uptake within these treatment settings. Although previous works have assessed how organizational leadership affects gains from TFW implementation [56,57,58,59], none were conducted among homeless-serving agencies.

### 1.1. Theoretical Framework

In keeping with recommendations by implementation researchers [60], Weiner’s organizational readiness for change theory [61] was used to better understand and explain the factors underlying the success or failure of implementation of evidence-based tobacco cessation efforts. Focused on systemic rather than individual readiness for change, organizational readiness for change is a multi-level construct that attends to the shared capacity among organizational members to implement a change. This theory was selected as particularly relevant given the focus of the Taking Texas Tobacco Free (TTTF) program on enacting systemic changes across multiple levels to affect transformations in organizational culture. The key determinants comprising organizational readiness for change include: (1) change commitment (valence): how much organizational members collectively value the proposed change, and their resolve to take on the changes involved in implementation; (2) change efficacy: the belief organizational members have in their skills, resources and capacity to collectively implement the various tasks required to operationalize the change; and (3) contextual factors: the degree to which broader conditions such as the structure, culture and resources impact organizational members’ capacity or willingness to implement the change [61,62]. Weiner conceptualizes “readiness” as a psychological construct that encompasses organizational members’ ability (preparedness) and willingness to implement change. While organizational structural factors affect the capacity to implement change, they do not explicitly define “readiness”. This distinction between “readiness” and capacity underscores the generative power present in organization—wide commitment and efficacy that can be harnessed to creatively utilize organizational structures and resources to achieve change [61].

### 1.2. Study Aims

The present study focuses on understanding how several factors contributing to organizational readiness across the organization as a whole influenced the TFW program implementation rather than simply the impact of leadership. This study assessed the role of organizational readiness in the implementation of a TFW program across three participating homeless-serving agencies in Texas. The aim of this work was to qualitatively examine the enablers and inhibitors of implementing a comprehensive TFW program within agencies serving adults experiencing homelessness to reduce the tobacco-related cancer risk behaviors among this population.

## 2. Materials and Methods

### 2.1. Intervention: Taking Texas Tobacco Free

Taking Texas Tobacco Free is a multi-component, evidence-based TFW program focused on building organizational capacity to treat tobacco dependence by targeting known implementation barriers across multiple levels—organizational, provider, and client [25,31,32,33]; as such, it is a comprehensive program. The program relies on the identification of a program champion—an agency provider or manager who is tasked with overseeing the implementation. This was a volunteer position that was not additionally financially compensated. The program champion was selected via consultation between the TTTF implementation team and agency leaders on the characteristics and requirements of the role. Built on an academic–community partnership, the TTTF program components include: (1) organization-wide TFW policy implementation and enforcement; (2) staff education on the harms of tobacco use; (3) specialized program champion and provider training on regularly assessing and treating tobacco dependence using brief evidence-based interventions (e.g., 5As; referral to quitlines), and delivery of these interventions, with additional training sessions including motivational interviewing [63] and a train-the-trainer program [64,65]); and (4) provision of cessation resources, including NRT–gum, lozenges, and patches—as well as hands-on guidance (i.e., technical assistance) from TTTF team members throughout the implementation process.

While a total policy ban (i.e., prohibition of the use of tobacco and other nicotine delivery products throughout the worksite) is standard TTTF programming, a compromise was made to permit a partial TFW policy (i.e., to allow designated tobacco use areas) within homeless-serving agencies to reduce the barriers to program participation. Shelter administrators have justifiable concerns regarding the safety of clients, who may be forced to leave the property to smoke under a total ban, which leaves them more exposed to victimization and violence [23,66]. Despite the implementation of a partial rather than a total TFW policy in participating homeless-serving agencies, TTTF is considered a comprehensive intervention, as it aims to reduce tobacco-related cancers through targeting system-level changes that promote change in organizational culture, policies, and practices on treating tobacco dependence [29,30,32,67,68,69]. A mixed methods, formative evaluation process was used to guide and tailor the implementation to individual agency needs.

### 2.2. Study Design, Participants, and Recruitment

The current study reports on the findings from the qualitative component of a mixed methods study focused on adapting, implementing, and evaluating a comprehensive TFW program within 3 homeless-serving agencies serving 3 counties in Texas. While the baseline quantitative data focused on organizational and client characteristics, demographics, and organizational readiness are reported here, these data are included to elucidate and contextualize the qualitative data that are informed by the organizational readiness for change theory.

Funded to work with 2 or more homeless-serving agencies within Texas, potential partners were identified from publicly available websites and lists, reaching out to pre-existing networks, as well as attending professional conferences. Community agencies that served those experiencing homelessness were targeted for recruitment and included faith-based organizations and residential as well as non-residential programs. Agency leaders were sent email invitations and information on program specifics and benefits. A cover letter was sent to agency leadership informing them of the study details and to obtain informed consent. After written consent for program participation was obtained from 3 agencies in the form of a memorandum of understanding, recruitment was closed. The 3 homeless-serving agencies joined the project at roughly the same time, within months of each other, and the active implementation phase of the TTTF program was expected to generally last 6–9 months. However, the research team transferred to a different academic institution in October 2022, at which point the grant was paused for a period of 3 months while financial and regulatory processes were established at the new institution. This hiatus in the grant timeline resulted in an extension of the active implementation period for the participating agencies. Table 1 provides details on the timeline and implementation of the program components by agency.

There were meaningful differences as well as similarities between the 3 agencies participating in this program (Table 2). While all were small-scale, low-resourced, non-profit agencies that served those experiencing homelessness, their organizational structures were distinct. Agency 1 was a non-profit, charitable faith-based outreach organization, which was administered by the 2 founders. Agency 1 relied on volunteers to assist with food distribution and made referrals for an estimated 1000 unique individuals annually for housing support and for mental health, substance use and tobacco use dependence treatment. Agency 2 was a community-based outreach center, with a limited staff of 3, which focused on transitioning those experiencing homelessness into permanent housing; the services offered included a food bank and case management assistance to 180 unique clients annually (e.g., to apply for employment and benefits, to obtain medical care). Agency 3 was a women’s shelter that was distinct in providing a residential program in which women paid minimal rent at subsidized rates for an apartment for themselves and their children. With a clinical staff of 12, this agency supported 175 women annually in transitioning to stable housing and employment through consultations with social workers, training in money management, life and parenting skills, and employment coaching and assistance. As such, the services provided at these agencies varied, with the women’s shelter being capable of delivering sustained assistance to their clients. The primary mission of these agencies was to provide clients with food assistance and securing stable shelter, along with administering to their spiritual needs for the faith-based organization. These organizations served those experiencing homelessness, most of whom had been homeless for the past 5 years and who had high rates of substance use (60% alcohol use; 30% cannabis use) and mental health disorders (60% post-traumatic stress disorder) and tobacco dependence (30%), and prior histories with the criminal justice system (32.5%).

An exploratory, qualitative design using conventional content analysis of individual and group interviews was selected as most suitable to explore the perspectives and experiences of participants from the 3 agencies involved in the program implementation [70]. The interview participants (*n* = 13) were primarily providers (*n* = 10), and a few managers (*n* = 3), directly involved in the program implementation. Prior to study participation, the interviewers discussed the nature of the study and interview questions with the participants, who verbally consented. Additionally, the voluntary nature of study participation was discussed; the participants were informed that they could withdraw from the study at any time and could decline to answer any questions. Solicitations were made to our program partners for permission to conduct individual or group interviews with their clients. However, all 3 agencies declined to provide us with permission to interview their clients, a decision which we respected. Given that those experiencing homelessness are subject to high rates of victimization and violence, and subsequently, trauma, it is understandable that many agencies serving these populations adopt very protective attitudes toward those they serve [22,65]. Each participant was compensated with a $40 e-gift card for Amazon.

A constructivist framework guided this study, as we were interested in understanding the social processes and contexts informing the participants’ perspectives [71]. This framework was selected because it allowed us a better understanding of how the participants construct their social interactions and unique experiences as providers of services to those experiencing homelessness, and what they find meaningful. Given the small size of the participating organizations, with full-time staff ranging from 2 to 17, we sought to recruit a total population sample [72], a type of purposive sampling that includes the entire population being studied—in this case, all the staff (including providers and managers) who were involved in the program implementation at the 3 participating homeless-serving agencies. Interview participants were recruited through email communications with the agency program champion who was offered TTTF sponsorship for a 5-day Certified Tobacco Treatment Training [73].

### 2.3. Data Collection

The organizational and client characteristics of the participating agencies were collected through a baseline leadership survey focused on demographics, current TFW policies and procedures, as well as organizational readiness. The 24 organizational readiness items on the survey were pulled from the Organizational Readiness to Implement Change (ORIC) questionnaire [74], which assesses organizational needs and characteristics regarding knowledge, practice, skills and readiness to implement change. Minor adaptations to the items were made (e.g., use of “agency” instead of “organization”) by the research team to best fit the delivery setting (i.e., homeless-serving agencies) in the present work. The ORIC includes 5 subscales with 24 items measuring change efficacy, change commitment, change valence and the resources and skills needed for change from an organizational viewpoint. The researchers selected this measure to assess the degree of organizational readiness or preparedness of the participating agencies to implement change within their organization. Leadership personnel at each agency were administered this measure pre-implementation and instructed to answer items reflective of how they perceived the entire workforce to feel about the upcoming TTTF implementation. The items include, for example: “People who work here feel confident that the organization can get people invested in implementing this change”; the responses were scored on a 5-point Likert scale ranging from 1 (disagree) to 5 (agree).

A total of 3 individual and 4 group interviews, consisting of 2–4 participants in each group, were conducted with the participants using a semi-structured interview guide between January 2022 and July 2023, lasting from 30 to 90 min. A pre/post design was used, which allowed data from pre-implementation interviews to inform the tailoring of the program to the specific needs and contexts of individual agencies to facilitate successful implementation. Given the COVID-19 safety considerations, these interviews were conducted live but virtually, using a videoconferencing platform. All the participants granted permission to audio- and video-record the virtual interview prior to participation. The development of the interview guides was informed by the research aims, the organizational readiness for change model [61], and prior implementation research on the factors impacting successful adoption of tobacco cessation programs within homeless-serving agencies [25,28,49]. Written field notes were also kept throughout the data collection on impressions about the participants’ responses and group interview dynamics and uploaded to Atlas.ti to inform the data analysis. The timeframe between the pre- and post-implementation interviews varied between agencies, ranging from 1 to 1.4 years. The interview guides were pretested and revised following 3 initial interviews. A cultural anthropologist and public health researcher (IML) trained in addressing tobacco-related cancers among subgroups disparately impacted by tobacco use conducted all the interviews. The IML specializes in health disparities research and has prior experience of implementing tobacco cessation interventions in healthcare organizations serving disadvantaged subpopulations. The researchers conducting the qualitative procedures had no prior relationship with the individuals participating in the study.

The pre-implementation interview questions focused on gathering baseline information about current tobacco-free policies, tobacco cessation services and assessments offered; reviewing various program materials (e.g., educational posters and brochures) to help develop and adapt materials to the specific needs and characteristics of the populations served (e.g., language, race, ethnicity, age, sexual orientation, sex, gender identity); prior tobacco treatment training received; expected challenges to and facilitators of program implementation; organizational members’ and leaderships’ attitudes toward TFW programs and smoking; unique agency needs and characteristics regarding program implementation; and any concerns about program implementation.

The post-implementation interview questions focused on understanding and assessing the implementation process and identifying barriers and facilitators to implementation. The questions also centered on any program adaptations made; how and why some implementation components were effective, while others were not; and any recommendations for program improvement.

### 2.4. Data Analysis

Quantitative data collected from the baseline demographic survey and from the ORIC questionnaire were analyzed using descriptive analysis and frequency counts.

All the interviews were audio-recorded and transcribed verbatim by a professional transcription service, anonymized and uploaded to Atlas.ti 9 (Atlas.ti, Scientific Software Development, version 9.1.6, Berlin, Germany, 2020) to organize the data analysis. Data from the pre-implementation individual and group interviews were analyzed first to tailor the intervention to local contexts and then compared across groups to understand the program partners’ baseline environment for addressing tobacco use. The post-implementation interview data were analyzed and compared to the pre-implementation results to understand any changes in attitudes, practices, policies, and knowledge regarding treating tobacco dependence among clients.

An inductive–deductive, or hybrid, approach was used for the data analysis. Starting with conventional content analysis [75], coding progressed iteratively, with 2 analysts (IML, AR), both cultural anthropologists and health disparities researchers, independently coding 6 transcripts inductively using constant comparison to develop a preliminary codebook. This analytic approach was selected because it allows for the coding of latent content and thus is well suited to understanding the complex factors and processes influencing participants’ experiences of program implementation. The codes were drawn directly from the data rather than being predetermined. Most codes were identified in the first 6 transcripts, with some added later in the refinement of the codes. The analysts met to discuss, refine and reconcile any coding discrepancies to finalize the codebook that was reapplied to all the data; the codebook remained open to refinement throughout the data analysis. Through iterative coding cycles, the analysts met regularly to continue category development using constant comparison to discern links between codes, combining codes into subcategories and categories. In conventional content analysis, the analytic process yields content categories rather than themes as the patterns identified in the final analysis [75]. The process of constant comparison served to refine the code and category development and their appropriateness, check for redundancy, and accurately account for the dataset [76]. A deductive approach was then used in which the inductively derived categories were viewed and compared by analysts to the organizational readiness for change constructs. In the last stage of the data analysis, analysts used the 3 organizational readiness for change constructs under which the initial subcategories were aligned and organized. The guidelines of the Standards for Reporting Qualitative Research (SRQR) [77] were followed in reporting this study (Table S1 File).

## 3. Results

### 3.1. Quantitative

#### Organizational Readiness to Implement Change

The results from the ORIC questionnaire (Table 3) indicate that, on average, the organizations scored highest on change efficacy, change commitment, and change valence (i.e., 4.62, 4.40, 4.80, respectively), demonstrating that, at pre-implementation, the program partners generally felt confident about their capabilities to implement the organizational changes required to address client tobacco dependence, valued this change, and were resolved to do so. The scores regarding knowledge and resources were slightly lower, on average (i.e., 3.56 and 3.75, respectively). Organization-wide training in treating tobacco cessation and provision of cessation aids, including NRT, were included in the comprehensive TFW program precisely to overcome these recognized barriers to treating tobacco dependence within these settings.

### 3.2. Qualitative Findings

To facilitate implementation, a formative evaluation process was used to tailor the program to the needs of our partners, e.g., adapting training sessions to the available time, adapting documents, writing up tobacco-free policies, including partial policies, and creating educational dissemination materials focused on the specifics of the populations served. As our study sample represented a relatively homogenous group, our research aims were narrowly defined, and as the interviews were structured following a semi-structured interview guide, the researchers are confident that data saturation was attained with 13 participants [78,79]. The data analysis yielded 10 subcategories organized into 3 main categories framed according to the organizational readiness for change constructs, change commitment, change efficacy and contextual factors (Table 4). From pre- to post-implementation, the homeless-serving agencies saw increasing or new challenges impact the program partners’ attitudes, wherein factors that initially served as program enablers (E) were reframed as inhibitors (I) at post-implementation (E/I). Thus, in Table 4, some categories serve as enablers and inhibitors, while others are simply enablers or inhibitors. Participants’ quotes, using pseudonyms, are shared to support the findings.

#### 3.2.1. Category: Change Commitment

##### Pre-Implementation

This category comprises the subcategories of *program buy-in/value*, *motivation to change, valuing benefits to clients* and *TFW policy support*. Each of these subcategories is an indicator of how open the organizational partners were to taking on the work necessary to implement change within their agencies. In the pre-implementation interviews, most participants stated that they valued the program highly; staff supported and were looking forward to the implementation, indicating significant *program buy-in*:

*Just really excited to be a part of this. I think that there’s a great opportunity to provide smoking cessation at [Agency 2] because about 80% of our population smokes. That’s why we’re excited to be here so we can offer that opportunity for our clients in the future.*

(Carlos, pre-implementation, Agency 2)

Staff were also willing to learn and make the necessary changes to implement tobacco cessation services in their agency. These changes included quitting smoking themselves, suggesting *motivation to change*:

*I think the staff would be willing to work with a smoking cessation program and get onboard with the program and become those leaders that other smokers can look up to…I’ve always believed in leading by example. If we want to have a smoke free facility, we have to lead by example.*

(Frank, pre-implementation, Agency 2)

The *benefits to clients* from implementing a TFW program were apparent to participants and were cited as a key motivation for joining the initiative:

*Even if we can get one person to stop smoking, I think that’s already a victory for us, but once we get that first person, I mean, that’s our stepping stone and it’s going to encourage us to keep moving forward with the program.*

(Sam, pre-implementation, Agency 3)

Likewise, the participants saw the value of implementing a TFW policy. They recognized that creating a tobacco-free environment would support clients in their attempts to quit smoking and stay tobacco-free:

*It’s* [TFW policy] *being an encouragement to help them with that path [tobacco cessation], to be able to be that support as well.*

(Sue, pre-implementation, Agency 3)

##### Post-Implementation

During the post-implementation interviews, the participants’ *valuing* of the program and *motivation to change* had significantly diminished. So much so that Agency 1 withdrew from active program participation (but not data collection), citing inability to continue with the implementation given existing priorities that were more highly valued:

*We just bit off more than what we could chew. We’re doing the food distribution. Of course, we do our ministry. That’s our first thing that we do…We just weren’t able to commit no more.*

(Juan, post-implementation, Agency 1)

Both other agencies experienced high staff turnover in which the program champions overseeing the program implementation left the agency or the position. As no other staff member stepped in immediately to lead implementation efforts, the program was stalled for several months in Agency 3 until a program champion was selected and ceased altogether in Agency 2:

*The person that brought the program to our organization left shortly after we started it. As far as me, myself, I never implemented any part of it…Then the person that stayed in charge of that, which was the other big manager, he’s no longer here with us.*

(Bianca, post-implementation, Agency 2)

However, the subcategories, *valuing benefits to clients* and *TFW policy support* both remained enablers throughout the implementation process and continued to be supported by participants:

*I still see it [TTTF program] as a huge need here in our area, because it’s helping people and it’s an addiction… “Wow. This is something that we need and it’s not here in our community”. One day, maybe when we have full staff and we’re able to do things, I would love to reevaluate again, but right now, we’re just day by day.*

(Juan, post-implementation, Agency 1)

*Nobody is allowed to smoke here. We have signs all over the place, which you guys provided. We do have them all around like inside in our patio area and everything. So, some of our clients that do smoke go outside the premises to smoke.*

(Rosa, post-implementation, Agency 2)

#### 3.2.2. Category: Change Efficacy

##### Pre-Implementation

Change efficacy comprises the subcategories of *valuing training to treat tobacco use* and *perceived fit with organizational culture*. During pre-implementation, the program partners highly valued receiving training on treating tobacco dependence as being essential to effectively addressing client tobacco use, as well as educating them on its harms. Additionally, addressing tobacco dependence was perceived as being aligned with the organizational culture and mission:

*I feel that we need some training. I think we need to educate. You’ll have a higher response if you educate* [clients] *and maybe a higher commitment than just picking up a patch today and never coming back again. We want buy-in, but they’re not going to buy-in if they don’t know… For example, people don’t know that there’s a connection between drug and alcohol addiction and tobacco addiction…That’s our biggest focus, is to help, but there is a lot of need. There’s a lot of poverty in our area. That’s why I believe in this program because I feel like it just goes hand in hand with what we do.*

(Juan, pre-implementation, Agency 1)

Another program partner stressed the importance of the specialized training provided to program champions in implementing tobacco cessation services within their organization, recognizing it as foundational to her being able to educate other providers in addressing tobacco dependence. She also spoke of how supporting their clients in tobacco cessation aligned with the theoretical model that this agency adopted in assisting those they served:

*I feel with the training that I’m going to get and the education that I’m going to provide our team when I return back from that week-long training, I’ll be able to coach different strategies on how to motivate individuals because we use a solution focused based theoretical model here… So, using [tobacco cessation services] in the strengths based approach and just really working with our families, just to support them in any way possible, we did think it was a good avenue that we should explore to provide that assistance for our families.….I believe it will help us be unified in the support that we provide our families.*

(Jade, pre-implementation, Agency 3)

##### Post-Implementation

At post-implementation, the partners’ perceptions of the *value of training* and *fit with organizational culture* were mixed. Incorporating tobacco education into new staff and annual training sessions was an implementation program aim/expectation that none of our program partners instituted (save a single new employee training session delivered to three employees that was provided by the first program champion at Agency 2). Agency 2 abandoned the program altogether after the three consecutive program champions left the organization rather than choosing to continue with the implementation:

*I never did anything with the program. It was mostly [Carlos and Frank] the ones that were in charge of that.*

(Bianca, Agency 2, post-implementation)

Although Agency 3 also did not integrate tobacco education into regular staff training sessions, the program champion did modify the NRT education materials and tracking documents to facilitate use by providers, providing them with ongoing training on the delivery of this evidence-based practice:

*I went through quite a bit of a process to break it down and make it usable for our case managers and not require them to go through different trainings because I know how taxing their job is…they rarely ever have time to bring other things into those case management meetings that they have weekly…I just wanted to make it as straightforward as possible for each of them and take on that time-consuming part of it myself.*

(Susan, Agency 3, post-implementation)

The *fit with organizational culture* is a key indicator of the perceived value of the program and its relative priority within the partner organizations. At post-implementation, there was a significant reframing of this construct due to shifting and competing priorities:

*As far as the case managers trying to do something with the clients regarding the program, like I said, our caseloads are extremely high so there’s just no way. [We serve] people that are probably like on the verge of being homeless, that number has increased and right now we are working very closely with the housing authority…they have a lot of needs.*

(Bianca, Agency 2, post-implementation)

#### 3.2.3. Category: Contextual Factors

##### Pre-Implementation

Although, as staff of historically low-resourced agencies, the participants were aware of the likely *resource* barriers, they still expressed a willingness and resolve to work with potential obstacles as necessary to implement the program, indicating that *resource* availability, in and of itself, did not deter their readiness to implement the program:

*That’s* [operational funds] *the only factor that we face as a challenge. We’ll find a way to put it out there where we can and make it happen. We are willing. We both are willing, and our team is willing…it’s* [TTTF program] *something to help their addiction. Is it going to take work? Yes. What doesn’t take work?* [Laughter]

(Maria, pre-implementation, Agency 1)

The participants initially related the importance of *leadership attitudes* and support for TTTF as a key driver of program implementation:

*Yes, I think it* [treating client tobacco dependence] *is a priority for leadership because that’s who brought it on and they wanted to provide the opportunity to our clients…so, we did think it was a good avenue that we should explore to provide that assistance for our families.*

(Donna, pre-implementation, Agency 3)

##### Post-Implementation

At post-implementation, attitudes toward the availability of *resources* had changed significantly. When asked what contributed to their not being able to implement TTTF, the program partners cited a lack of financial, time, organizational and personnel *resources* as impeding the program implementation:

*The caseload* [Laughter] *to be honest with you. I’ll give you an example. From January to March of this year, I actually had 158 clients that I assisted. Right now, my caseload is over a hundred… We’re a very small organization. I think there’s only like 20 employees. Two case managers.* [Laughter] *So, yes, it’s been kind of hard.*

(Bianca, post-implementation, Agency 2)

Leadership attitudes also significantly shifted from pre-implementation support, with providers and leaders alike communicating waning capacity and drive to continue with the program implementation. For example, this center leader stated:

*It’s just me and Maria and then our volunteers. I can’t do something like that, and it’s like I don’t have a volunteer to help me with the program… So, that’s why the* [program] *took a hit. We just weren’t able to commit no more.*

(Juan, post-implementation, Agency 1)

##### Contextual Factors Consistently Inhibiting Program Implementation

Two contextual factors served as persistent impediments to implementing the TTTF program throughout the implementation period: *COVID-19*-*related issues* and *staff turnover*. For these already under-resourced community agencies, COVID-19 resulted in the suspension of many services to agencies assisting those experiencing homelessness during the height of their need. During this same period, the federal government made grant funds available to businesses and organizations adversely impacted by COVID-19 that could demonstrate need. The media widely reported that many of these grants were awarded to wealthy businesses rather than low-resourced organizations that were in most need of these funds, which particularly frustrated our program partners:

*COVID hit. Everything went crazy. That* [services] *got interrupted because of COVID, people lost their jobs and you’re on the streets…During COVID the world stopped, okay? It’s really hurting the homeless—the funding came out of the woodwork, but some of those requirements are still there….It was hard enough to assist these folks, we have COVID and you’re throwing millions at millionaires, that have businesses, yet you find every opportunity to not help those that really need the help.*

(Carlos, pre-implementation, Agency 2)

*COVID hit hard here, a lot of families here lost a loved one, but there’s no bereavement center around this area and not everybody’s at the church either. But we did COVID packages for those who are positive. They contact us. They let us know they’re positive, and then we have team members who take them food, water, hygiene needs and everything. We got COVID and we didn’t give food this past month. We all got sick.*

(Maria, pre-implementation, Agency 1)

Likewise, high leadership and *staff turnover* at these homeless-serving community agencies was a constant inhibiting factor both pre- and post-implementation due to the lack of consistency in the TFW policy adoption and procedures, as well as the loss of in-house knowledge on treating tobacco dependence when trained providers left:

*There has been a turnstile of directors and staff here at* [Agency 2] *for a couple of years so there wasn’t very much consistency in policy, rules, and that kind of a thing. If clients choose to smoke, they have to step outside… I don’t even think we have it* [TFW policy] *that specific. It’s basically you’re not allowed to smoke inside… With the proper training, of course, we would be able to offer a better service, but until now, we have not been providing any smoking cessation program to our clients, and it’s due to the lack of training*

(Carlos, Agency 2, pre-implementation)

*The person that brought the program to our organization left shortly after we started it…I know the other case manager was providing some information but since he is no longer here…we have tried to look through his paperwork and to be honest with you, as far as like paper trail or anything that he was doing, we have not found anything that he did.*

(Bianca, Agency 2, post-implementation)

## 4. Discussion

The current study sought to understand and identify the factors enabling and inhibiting implementation of a comprehensive TFW program, called TTTF, within homeless-serving agencies within the US [25]. This work is novel in viewing the factors and processes contributing to the successful implementation of a TFW program through the lens of “organizational readiness”. The concept of “organizational readiness” outlined in the organizational readiness for change [61] theory was selected as an applicable and effective framework to explore the factors that contribute to or impede an organization in implementing a comprehensive evidence-based tobacco cessation intervention in terms of collective readiness, motivation and capacity. The final qualitative analytic subcategories were mapped onto the organizational readiness for change constructs, change commitment, change efficacy and contextual factors, to frame, guide and categorize the findings from the group and individual interviews with agency providers and managers. As a multicomponent, complex intervention, the TTTF program focused on organizational change and building organizational capacity within homeless-serving agencies to address tobacco dependence and reduce tobacco-related cancers among those experiencing homelessness. The different factors identified in this study as enabling or inhibiting organizational change were all encompassed within this model of change. We identified 10 subcategories that described the factors serving as enablers or inhibitors, or both, over the course of constantly shifting contextual influences throughout the program implementation. 

At pre-implementation, the participants at the three homeless-serving agencies expressed significant change commitment, as evidenced by strong program buy-in, motivation to change, valuing program benefits to clients, and support for implementation of a TFW policy. Initially, the providers were excited about the program implementation and even expressed determination to overcome such barriers as staff tobacco use by making personal—and encouraging—staff quit attempts to lead clients by example. This is in keeping with the TTTF program, which encourages extending tobacco cessation services and resources to staff to promote and facilitate organizational support and program adoption [57]. The participants also recognized the great need for a tobacco cessation program to address the high tobacco use rates among their clients. While the participants continued to value the benefits of adopting a TFW program and policy throughout the program implementation, their acceptance of and support for treating tobacco use as well as their motivation to adopt changes waned significantly at post-implementation. This shift in attitude was mainly attributed to changing contextual factors. However, this finding may suggest that supporting a comprehensive TFW program and the indisputable benefits that it provides to clients, staff, and homeless-serving agencies in preventing tobacco-related cancers is, *theoretically*, quite distinct from valuing and appreciating the effort and persistence needed to implement organizational change in *practice*. Particularly so given the long-standing pro-tobacco culture [21,40] within homeless-serving settings, in which historically, providers not only failed to treat tobacco use among clients but received charitable contributions from the tobacco industry along with free tobacco products to encourage brand promotion. Other studies have noted that high tobacco use among staff at homeless-serving agencies, as within substance use treatment, is a common barrier to addressing client tobacco use [20,80]. In fact, a survey of clinicians within a national network found that 15% of respondents reported providing clients with tobacco as a means of building rapport, trust and adherence, with one-third stating they knew colleagues who engaged in this practice [26].

Despite recommendations from national practice guidelines for providers [50,81] to assess and deliver brief treatment for tobacco dependence at every opportunity, provision of these services to those experiencing homelessness remains limited [49]. In a qualitative study on quitting smoking among those experiencing homelessness who were highly dependent upon tobacco and other substance use, the participants reported making independent quit attempts, i.e., going “cold-turkey”, and not having received any provider support or even being actively discouraged by providers from addressing their tobacco dependence [27]. The providers cited concerns that concurrent tobacco and substance use treatment would jeopardize substance recovery, a common but fallacious belief that research has soundly disproven [82,83]. It is precisely due to the persistence of such organizational biases, misconceptions, and norms regarding treating tobacco dependence among those experiencing homelessness that organizational change is needed to integrate provision of tobacco dependence care into routine practice within these settings. Changing provider attitudes, behaviors and practices is essential to engendering organizational change regarding addressing tobacco dependence, particularly as tobacco use has long been normalized within these homeless-serving agencies [84,85] and other similar settings serving disadvantaged groups, and these misconceptions are communicated to and internalized by clients. Even so, research attests that those experiencing homelessness are motivated to quit tobacco use [86] but face multiple challenges; key among these is the lack of provider support in quitting. This population infrequently accesses healthcare, given the myriad structural barriers they face in a for-profit healthcare system where medical coverage is primarily based on employment. Unfortunately, these individuals also are alienated from and mistrust traditional service systems, which further limits their opportunities for accessing much needed healthcare [87]. Additionally, this study was conducted in Texas, which in 2021, ranked highest nationally in uninsured rates at 20.5% in comparison to the 10.2% national average and is a state that has not adopted Medicaid expansion under the Affordable Care Act [88]. As such, the providers at homeless-serving agencies may be, in some cases, the main source of healthcare as well as housing services for these individuals. This affords providers with an invaluable opportunity to address their clients’ physical, mental and substance use disorders, including tobacco dependence, which is linked to the various cancers that are a key cause of death and disease within this population.

Fortunately, post-implementation, the program partners continued the implementation of their TFW policy, valuing the benefits it provided to clients and staff. Adoption of such policies is primary for tobacco cessation efforts and clearly indicates a lack of support for tobacco use within these settings. This is an important step toward changing the historical organizational attitudes and culture around tobacco use as an accepted social norm. As the primary objective of homeless-serving agencies is to provide much-needed services to this population, concerns that adoption of tobacco-free policies within these settings would drive clients away often inhibit their adoption. However, people experiencing homelessness who smoke and have substance use disorders have reported that adoption of environmental TFW policies was effective in managing their tobacco use [27,33] and research indicates that occupancy rates may not be affected by adoption of these policies [24]. The adoption of tobacco-free policies within these and other settings has proven effective in reducing tobacco use [41,89,90].

Change efficacy was related to the partners valuing being trained to treat tobacco dependence and the perceived fit of the program with the organizational culture. Pre-implementation, the program partners reported that receipt of training in treating clients’ tobacco dependence was crucial to assisting them in making quit attempts and were enthusiastic about being trained. Training was also viewed as essential to providing client education on the harms of tobacco use. This assessment is in keeping with multiple studies focused on treating tobacco use among the various disadvantaged subgroups with the highest smoking rates that report providers’ lack of knowledge on treating tobacco dependence is a primary obstacle to providing their clients with cessation services [91,92,93,94]. Likewise, the TTTF program was viewed by our partners as being aligned with the mission and culture of their organization, which involved supporting the overall health of their clients—i.e., addressing physical, mental (including substance use), and spiritual needs—which facilitated agency-wide buy-in and adoption. However, at post-implementation, Agency 1 withdrew from the program altogether and of the two remaining partners, Agency 2 had abandoned implementation after the departure of three consecutive program champions and Agency 3 had paused implementation for half a year until a new program champion was identified. Training was recognized by the program partners as essential to delivering tobacco cessation services to their clients, and all received the specialized provider training on treating tobacco dependence provided by TTTF staff. However, only one of the program champions, who soon left Agency 2, took the opportunity to participate in the five-day Certified Tobacco Treatment Specialist training sponsored by the TTTF program. Additionally, even though in Agency 2, one of the three providers that participated in the train-the-trainer program continued at the agency, this provider did not train others in, or provide clients with, tobacco cessation services. At post-implementation, both of these factors initially perceived as enablers later ceased to function as such, being perceived as inhibitors at Agency 2, as they no longer served as a shared value and belief in organizational members’ collective capability to change [61], due to contextual changes. At Agency 3, however, the new program champion initiated modification of the NRT educational materials and distribution tracking documents to facilitate their use by providers and continued providing training on this evidence-based tobacco cessation practice.

Overall, contextual factors were described by the program partners as underlying the reframing of the factors that were previously perceived as enablers into inhibitors to program implementation. The various contextual factors are related to resources in different ways, be they financial, leadership or supportive, knowledge, staff, time and COVID-19 resource issues. The partners cited excessive caseloads and thus, limited time, due to loss of staff as impeding any chance of addressing tobacco dependence with clients. However, there are brief interventions, such as the 5As [50], recommended by clinical guidelines as an effective evidence-based tobacco cessation practice that could be integrated into delivering other client services, such as during substance use counseling. The providers at the partner agencies were trained on making referrals to the quitline and the 5As as part of the training delivered by TTTF staff on treating tobacco dependence among those experiencing homelessness. The perception that delivering tobacco cessation interventions to clients within these settings is time-consuming is not accurate, as a quitline referral is quick and using the 5As can take less than 3 min [95], and speaks more to the fact that the providers feel overwhelmed, under resourced and unsupported.

While some partners saw changes in leadership, including loss of consecutive program champions who led the implementation efforts, others saw a diminished focus on treating tobacco dependence within the context of dwindling resources and contending priorities, also noted by other research [41]. Lack of resources—funds, time, staff, training—needed to attend to the complex, multiple needs of this population is chronic within these settings and often cited as a barrier to implementing change [87]. Organizational stressors often necessitate the “triaging” of services within these homeless-serving agencies, and as with substance use treatment centers, contribute to a devaluing of treating tobacco as a serious addiction [13,43,44]. Two contextual factors functioned as consistent inhibitors to integrating tobacco dependence services within these agencies, COVID-19-related issues and staff turnover, for the duration of the program. COVID-19 posed challenges for the partner agencies throughout the program implementation, contributing to site closures, staff turnover, and decreased finances, while also increasing the numbers of those experiencing homelessness locally and thus demand for services. Of the different variables measured by the ORIC questionnaire, the two with the lowest scores were “knowledge” (mean 3.56), and “resources” (mean 3.75). Despite their acknowledged resource limitations, at pre-implementation, the program partners were eager to participate in the program and reported high levels of change commitment and change efficacy, being willing and capable of overcoming capacity challenges to implement the program.

However, at post-implementation, changing contextual factors had overwhelmed our partners to the extent that they either explicitly withdrew from (Agency 1), abandoned (Agency 2), or paused (Agency 3) program implementation. All of our program partners, like many community healthcare organizations, were under-resourced financially, were chronically understaffed, had excessive workloads and were decidedly stressful work environments, which contributed to the high staff turnover rates, as confirmed by other studies [87,96,97]. The larger context contributing to the resource challenges experienced by our program partners is the lack of state support for addressing tobacco dependence within homeless-serving agencies as well as healthcare organizations state-wide. The American Lung Association’s State of Tobacco Control evaluates state-level implementation of policies and laws proven effective in eliminating the death and disease caused by tobacco use by assigning letter grades. This study was conducted in Texas, which was rated F in 2024 on most evaluation categories regarding limited tobacco prevention and cessation funding; smokefree air, i.e., extremely limited provision of state smoking restrictions; limited tobacco taxes; and no restrictions on flavored tobacco products. The one exception of the rating *D* was on access to cessation services, which is an improvement from *F* in 2023 [98], in that all seven cessation medications are covered by Medicaid and state health plans, but coverage by private plans is not mandated.

The organizational readiness to change theory holds that “readiness” describes a psychological and behavioral willingness to take on change, based on change commitment and change efficacy, which can be determined or actualized by contextual factors or “capacity” for change. The study findings appear mixed regarding the applicability of the organizational readiness to change theory and its ability to account for readiness to change. In the case of Agency 1 and 2, both ceased to implement the program largely due to capacity or contextual factors, which ultimately affected their change efficacy or belief in their capabilities to execute the tasks required for change. Whereas contextual changes led Agency 3 to pause the implementation, hiring another program champion altered their change efficacy, allowing for reinstatement of the program. As such, the study findings support “readiness” as a psychological rather than a structural construct, in that the changes in contextual factors in non-implementing centers were related to alterations in change efficacy resulting from contextual factors and “capacity”; hence, organizational “readiness” was affected. The different constructs of the organizational readiness to change theory work synergistically to affect or impede organizational change. As such, changes in one part of the model can engender changes throughout the system that can render program implementation unfeasible. Yet, in the example of Agency 1, which lost the capacity to implement the program at post-implementation, the program partners continued to value TTTF and expressed an interest in future implementation if and when resources became available, which indicates continued change commitment.

### Study Limitations and Implications

This study focused on the enablers and inhibitors of implementation of a comprehensive TFW program within homeless-serving agencies in Texas. Given the important influence that state tobacco cessation policies, laws and funding exert on the operation of these community agencies, our findings may not be applicable or transferable to similar settings in other geographic locations. While this study identified various enablers and inhibitors of implementing a TFW program within homeless-serving agencies, our findings are not meant to be exhaustive; other studies may report different findings. This study was conducted during the height of the COVID-19 pandemic, which adversely affected the agencies’ capacity for program implementation. Additionally, the research team transferred to another academic institution, which lead to a 3-month hiatus in the grant and implementation of the project by our program partners. However, by this time, Agency 1 had already withdrawn from active program participation, and Agency 2 had ceased program implementation without leaving the project; both agencies continued contributing data to the study. Agency 3 underwent its own internal hiatus in program implementation due to being without a program champion for approximately 6 months, which overlapped with our 3-month grant pause. Another limitation is that the program partners were very protective of their clients and did not allow us access for interviews. Presenting clients’ perspectives along with those of providers would have enriched this study and permitted us a different perspective on their needs and preferences regarding tobacco cessation. Future studies should present the views of clients and providers that would afford a more comprehensive and accurate view of the enablers and inhibitors of program implementation within these settings to inform the development of tailored tobacco cessation interventions. The research literature indicates that provider and client reports on the enablers and inhibitors of the adoption of tobacco-free programs often contradict each other, e.g., regarding client interest in quitting and the acceptability of such programs [24].

The use of a qualitative approach was a strength of this study. As this method is exploratory it is particularly suitable for filling knowledge gaps, given the lack of research in the factors affecting successful implementation of tobacco cessation interventions within these settings. The use of qualitative methods also provided a real-world view of the experiences and perceptions of the program partners in delivering and implementing tobacco cessation services. Such understanding is foundational to any community-based participatory research approach required to respond to the particular needs of people experiencing homelessness for adapted evidence-based tobacco cessation programs, along with assessment of the program partners’ organizational readiness to implement change [99]. While this study employed a hybrid, or inductive–deductive, analytic approach, our initial coding was conducted inductively, in which our subcategories were directly drawn from the data. The final stage of the analysis used a deductive coding approach based on the predetermined organizational readiness to change constructs to organize the inductively derived subcategories. This hybrid approach limited the risk of missing the complexities within the data commonly associated with starting with predetermined, deductive coding.

The number of people experiencing homelessness within the US has been growing steadily since 2017, with a 12% increase from 2022 to 2023 [5], impacting ethnic, racial and gender subgroups the most [100]. While homeless services have been expanded, they are not capable of meeting the increased demand from the rising numbers of this population. Program partners attest to being overwhelmed and chronically under resourced and in need of substantial assistance to continue their work. The adoption of tobacco-free policies is critical to supporting the clients of homeless-serving agencies in their cessation efforts through changing social norms around tobacco use, which was supported by the program partners and reinforced by prior research [90]. Homeless-serving agencies, like those within this study, need state and local assistance to support the enactment and legislation of tobacco-free policies to address tobacco-related health inequities among homeless and other disadvantaged groups. While population-based policies, such as the clean indoor air laws have been successful in decreasing tobacco use among the general population, they have had a limited impact on those experiencing homelessness. For this population, experts recommend passing state policies through building partnerships between homeless-serving agencies and tobacco control programs/advocates and state/local representatives [99].

The study findings indicate the need to educate and train providers on referral to national (e.g., for veterans) and state quitlines, as well as the use of the 5As. The 5As is a brief, evidence-based tobacco cessation intervention, which can be integrated into routine practice within homeless-serving agencies with limited time and high caseloads. Integration of tobacco cessation treatment into the continuum of care has been recommended as a priority intervention by experts [99]. While this program included the provision of a train-the-trainer package to build in-house expertise and thus stop the hemorrhaging of specialized knowledge on treating tobacco dependence, the staff turnover was so high at some participating agencies that trained providers were lost, or providers lacked the time to participate in training. Train-the-trainer programs could be adapted to suit the needs, e.g., available time and number of staff, of participating agencies, possibly delivering shorter and multiple training sessions to accommodate community partners. Given the important role of program champions in this study in leading the implementation efforts and driving organizational change, tobacco cessation interventions within healthcare centers serving those experiencing homelessness and other disadvantaged groups should ensure the integration of implementation champions. The identification and preparation of champions, along with train-the-trainer strategies, have been included and endorsed in the Expert Recommendations for Implementing Change (ERIC) study [101] on implementation strategies for use in combination or singularly in implementation practice. The findings indicate that additional research is also needed on understanding the barriers and facilitators to implementing tobacco cessation services within these settings to convince policymakers of the compelling need to address the high rates of tobacco use and tobacco-related cancers among those experiencing homelessness.

## 5. Conclusions

Despite the flexible implementation adapted to the needs of our different homeless-serving program partners, as well as the provider training on treating tobacco dependence and free NRT, the ever-changing contextual factors limited the full implementation of a comprehensive TFW program. Application of the organizational readiness for change model was useful in identifying the factors that enabled and inhibited program implementation within the participating agencies and alerted us to potential strengths and weaknesses within these types of organizations. Low-resourced agencies, like those participating in this study, require additional financial support, training, and innovation to recuperate from infrastructure challenges and to build the capacity needed to implement tobacco cessation care for clients. While the program partners identified specialized training on treating tobacco dependence as primary to addressing tobacco use among their clients, most lacked the time to devote to such training. The findings on the systemic challenges faced by homeless-serving agencies due to being under-resourced indicate a crucial need for state policy and funding support. This is particularly relevant in states such as Texas that have extremely high rates of uninsured people and limited safety-net options. Addressing tobacco dependence among those experiencing homelessness is challenging and requires dedicated effort and support in view of the multiple, complex needs of this population. The integration of tobacco cessation interventions within agencies serving the homeless is imperative to address the high tobacco-related cancer rates among this population, given that tobacco use is a modifiable risk factor linked to at least 13 cancers. Moreover, as individuals within this population may not seek assistance from traditional health service systems, these settings may be the primary site in which to address their tobacco dependence. The findings are novel in indicating that providers recognize and value the importance of tobacco cessation interventions that include tobacco-free policies to address the environmental and social influences contributing to tobacco use but are impeded from implementation due to resource limitations. Without the organizational capacity, e.g., structure, personnel, time, and funds, needed to operationalize change, the partners’ change efficacy decreased, as did their resolve to implement care for tobacco dependence. More research is needed to facilitate the integration of tobacco cessation services into these settings. The findings of this study will be instrumental in guiding future TFW program interventions, enabling the identification of agencies that are well-positioned to adopt such programs and facilitating capacity-building efforts to ensure their successful participation.

## Figures and Tables

**Table 1:** Implementation of the Taking Texas Tobacco Free (TTTF) program components and timeline by agency.

Agency and Timeline	TFW Policy	Staff Education (Last 12 Months)	Program Champion (PC)	Specialized Training (MI, TTS, T-t-T)	Resource Provision	Program Completion
Agency 1: Faith-based, non- residential; 12/21 to 9/22; withdrew from program implementation but not data collection	Adopted partial TFW policy with TTTF SI	No prior education on addressing tobacco use other than that provided by TTTF SI	One PC throughout implementation UI	Did not engage in any specialized training for treating tobacco use SI	NRT received but never distributed; NRT returned and redistributed to Agency 3 UI	No. Withdrew in month 9 due to competing priorities UI
Agency 2: Community outreach, non- residential; 12/21 to 8/22; abandoned implementation but not data collection	Adopted full TFW policy during TTTF SI	No prior education on addressing tobacco use other than that provided by TTTF; with the exception of 1 staff member SI	Three PCs left agency in succession over the course of ~6 months SI	First PC participated in TTS training; 3 providers (including first PC) engaged in T-t-T; 2 in MI UI	NRT received but never distributed; NRT was lost UI	No. After 3 PCs left, program was essentially abandoned UI
Agency 3: Women’s shelter, residential; 1/22 to 9/22; program halted internally from 9/22 to 4/23	Extended partial TFW policy during TTTF SI	No prior education on addressing tobacco use other than that provided by TTTF; with the exception of 1 staff member SI	Two PCs left position (1 left agency); without a PC for 6 months, so program was halted. New PC continued implementation SI	First PC participated in T-t-T for treating tobacco use SI	Received and distributing NRT to clients and employees SI	Yes. Once new PC was selected in 4/23, implementation continued SI

Notes: TTTF = Taking Texas Tobacco Free; TFW = tobacco-free workplace; PC = program champion; MI = motivational interviewing; TTS = tobacco treatment specialist; T-t-T = train-the-trainer; NRT = nicotine replacement therapy; SI = successful implementation; UI = unsuccessful implementation.

**Table 2:** Baseline characteristics of homeless-serving agencies participating in the Taking Texas Tobacco Free program (*n* = 3).

Agency	Clinics	Clinical Staff	Total Annual Unique Clients	Total Annual Contacts	Residential/ Outpatient
1. Faith-based	1	2	1000	3000	Outpatient
2. Community-Outreach	1	3	180	90	Outpatient
3. Women’s Shelter	1	12	175	10,000	Residential

**Table 3:** Pre-implementation scores on the Organizational Readiness to Implement Change questionnaire overall and by subscale collected from the leadership at the homeless-serving agencies participating in Taking Texas Tobacco Free (*n* = 3).

Variable	*n*	Mean	SD	Minimum	Maximum
ORIC efficacy	3	4.62	0.66	3.86	5.00
ORIC commitment	3	4.40	0.87	3.40	5.00
ORIC knowledge	3	3.56	0.19	3.33	3.67
ORIC resources	3	3.75	0.66	3.00	4.25
ORIC valence	3	4.80	0.35	4.40	5.00
ORIC overall	3	4.33	0.58	3.67	4.71

Note: *n* = 3 participating homeless-serving agencies. ORIC = Organizational Readiness to Implement Change [74]; items were rated on a 5-point Likert scale ranging from 1 = disagree to 5 = agree.

**Table 4:** Qualitative content analysis categories and subcategories of pre- and post-interviews with participants.

Category/ORC Construct	Subcategory	Description
Change commitment	Program buy-in/value (E/I)	Program buy-in indicates degree of staff’s valuing of treating tobacco use and the acceptability of doing so
	Motivation to change (E/I)	Staff’s motivation and willingness to adopt a tobacco cessation program into current workflows
	Valuing benefits to clients (E)	Perception of program benefits to clients, which drove making changes to implement tobacco cessation
	TFW policy support (E)	Support for adopting and enforcing the TFW policy is a crucial indicator of willingness to implement changes regarding addressing tobacco use
Change efficacy	Valuing training to treat tobacco use (E/I)	Training in treating tobacco use is a primary facilitator to increase skills and confidence to provide these services
	Perceived fit with organizational culture (E/I)	Compatibility with agency cultural values supports staff’s perception of being capable of treating tobacco use and its fit with existing systems and workflows
Contextual factors	Resources (E/I)	Availability of financial, time, organizational and personnel resources to implement tobacco use care
	Leader attitudes (E/I)	Leader attitudes can drive and support ushering in and implementing changes in treating tobacco use
	COVID-19-related issues (I)	COVID-19-related issues, i.e., agency closure and loss of financial, personnel and client resources impacted adoption of tobacco use care
	Staff turnover (I)	Relates to availability of staff that are trained, knowledgeable and capable of treating tobacco use

Note: Categories are organized according to the organizational readiness for change (ORC) constructs of change commitment, change efficacy and contextual factors [61], which are further delineated into 10 subcategories. The subcategories functioned as enablers (E) or inhibitors (I), or both, in that at pre-implementation some subcategories acted initially as enablers but over the course of implementation changed into inhibitors by post-implementation. TFW = tobacco-free workplace; E = enabler, I = inhibitor.

## Data Availability

Data are not publicly available due to privacy restrictions. The data that support the findings of this study are available from the first author, I.M.L., upon reasonable request.

## Supplementary Materials

The following supporting information is available online at: https://www.mdpi.com/article/10.3390/cancers16112162/s1, Table S1: The SRQR (Standards for Reporting Qualitative Research) checklist. Reference [77] is cited in the Supplementary Materials.
